# The nonlinear relationship between estimated glomerular filtration rate and cardiovascular disease in US adults: a cross-sectional study from NHANES 2007–2018

**DOI:** 10.3389/fcvm.2024.1417926

**Published:** 2024-11-22

**Authors:** Ce Zhou, You Zhou, Niannian Shuai, Jiaxiu Zhou, Xin Kuang

**Affiliations:** ^1^ Department of Anesthesiology, People's Hospital of Longhua, Shenzhen, China; ^2^ The Eighth Affiliated Hospital, Sun Yat-sen University, Shenzhen, China; ^3^ Shenzhen Second People’s Hospital, The First Affiliated Hospital of Shenzhen University, Shenzhen, China

**Keywords:** estimated glomerular filtration rate (eGFR), cardiovascular diseases (CVD), NHANES, nonlinear relationship, cross-sectional research, population study, multiple statistical analyses

## Abstract

**Background and aim:**

Estimated glomerular filtration rate (eGFR) is a key indicator of kidney function and is associated with numerous health conditions. This study examines the association between eGFR and cardiovascular disease (CVD) risk in a representative cohort of the US adult population.

**Methods:**

A cross-sectional analysis was conducted using data from the National Health and Nutrition Examination Survey (NHANES) from 2007 to 2018. The study included 31,020 participants aged 20 years and older. The eGFR estimates were calculated using the Chronic Kidney Disease Epidemiology Collaboration (CKD-EPI) equation. CVD was defined as a self-reported physician's diagnosis of congestive heart failure, coronary heart disease, angina pectoris, myocardial infarction, or stroke. To assess the association between eGFR and CVD risk, the study employed weighted linear regression and generalized additive models.

**Results:**

The study revealed a significant non-linear inverse association between eGFR and CVD risk, with a threshold effect observed at 99.3 ml/min/1.73 m². Below this threshold, each 10-unit increase in eGFR was associated with a 13% decrease in the odds of CVD (OR: 0.87, 95% CI: 0.84–0.90, *P* < 0.001). Above this threshold, no significant association was found between eGFR and CVD risk (OR: 1.04, 95% CI: 0.90–1.20, *P* = 0.60), indicating that further increases in eGFR beyond this point were not associated with additional cardiovascular benefits. Subgroup analyses revealed significant interactions for eGFR categories, anemia status, and ratio of family income to poverty (PIR).

**Conclusions:**

This study shows that there is a non-linear relationship between eGFR and CVD risk in the US adult population. The study found evidence of a threshold effect. These findings emphasize the importance of monitoring and managing CVD risk factors in individuals with reduced kidney function, especially those with eGFR values below the identified threshold. The relationship between eGFR and CVD risk varies across different subgroups, particularly in relation to eGFR categories, anemia status, and socioeconomic factors.The results provide valuable insights for developing targeted CVD prevention and treatment strategies based on kidney function status.

## 1 Introduction

The Estimated Glomerular Filtration Rate (eGFR) is a crucial tool for evaluating renal function. Chronic kidney disease, which is characterized by low eGFR or albuminuria, affects around 14% of adults in the United States (1). The 2017 Global Burden of Disease Study reported 697.5 million cases of all-stage chronic kidney disease (CKD), indicating a global prevalence of 9.1%. Additionally, 1.2 million people (95% uncertainty interval, 1.2–1.3) died from CKD that year (2). eGFR values reflect the rate at which the kidneys filter blood and are expressed in ml/min/1.73 m^2^. Equations that consider factors such as serum creatinine, cystatin C, age, and gender are commonly used to estimate eGFR. Variability in eGFR has been linked to long-term mortality (3), and abnormal eGFR levels have been associated with various conditions such as sickle cell disease (4), Parkinson's disease (5), cardiovascular disease (6), chronic kidney disease severity (7), sleep duration (8), and liver cirrhosis (9).

Cardiovascular diseases (CVD) refer to a range of disorders that affect the heart and blood vessels, such as ischemic heart disease, stroke, and others. These conditions are significant contributors to global mortality (10). The risk factors for CVD are diverse and include hypertension, dyslipidemia, and other related conditions (11). In 2019, there were an estimated 523 million cases of CVD globally, resulting in 18.6 million deaths, which accounted for 32.8% of all deaths, and 393 million disability-adjusted life years lost (12). Cardiovascular disease (CVD) presents a significant global public health challenge. Preventing and controlling CVD demands a collaborative effort from all sectors of society.

In addition, the impact of eGFR on cardiovascular outcomes has been studied in different populations. For example, in South Asian individuals, different eGFR categories were associated with risk of all-cause mortality, incident heart failure, and atherosclerotic cardiovascular disease (13). In addition, a study of young patients with kidney failure found that years spent with eGFR below a certain threshold were predictive of vascular stiffness, highlighting the cumulative cardiovascular damage from prolonged exposure to kidney dysfunction (14). Furthermore, a study of living kidney donors found an inverse relationship between eGFR and cardiovascular risk, indicating the importance of kidney function in cardiovascular health (15).

Although some studies have shown an association between glomerular filtration rate and CVD, research based on the U.S. population is relatively limited. NHANES is a nationwide survey with a sample covering a wide range of races, ages, and gender in the United States. This representativeness can enhance the generalizability and extrapolation of research findings. Leveraging the unique advantages of NHANES data, we conducted this study to comprehensively assess the relationship between eGFR and CVD risk and to explore how various demographic factors may influence this association. This analysis is intended to inform the development of more targeted strategies for CVD prevention and treatment.

## 2 Methods

### 2.1 Study design and population

Data for this study were obtained from the National Health and Nutrition Examination Survey (NHANES) database, covering the period from 2007 to 2018. NHANES is a continuous survey that uses a sophisticated multistage probability sampling strategy to select a representative sample of the U.S. population. This approach makes it possible to assess the health and nutritional status of adults and children in the United States. The survey data are publicly available online for global data users and researchers at www.cdc.gov/nchs/nhanes/.

**Figure 1:**
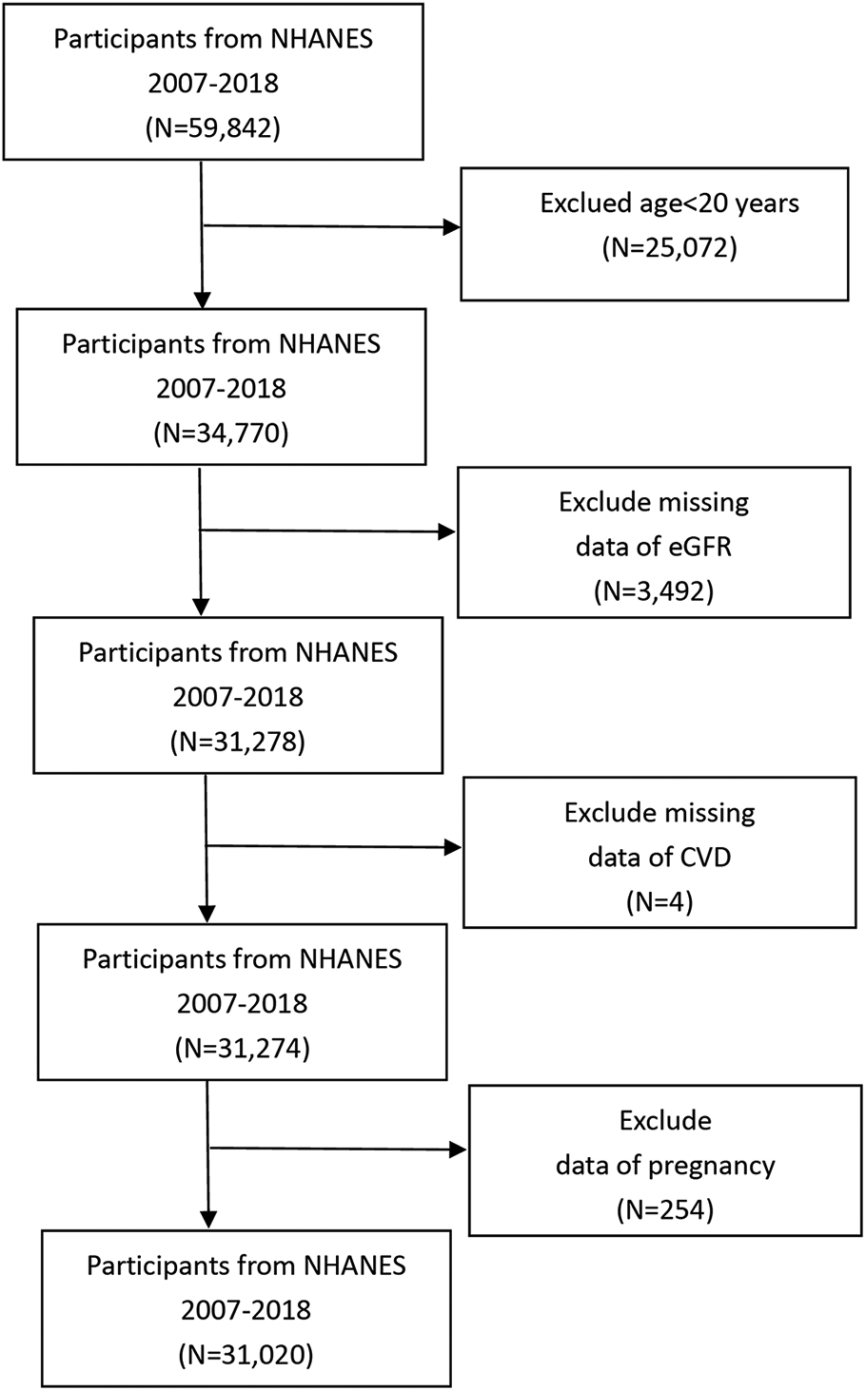
Flow chart of the process for selecting samples.

In this study, we conducted a cross-sectional analysis using data from NHANES (2007–2018), focusing on individuals aged 20 years and older, a total of 34,770 participants. Participants younger than 20 years were excluded (*n* = 25,072). Participants with incomplete eGFR data were also excluded (*n* = 3,492), resulting in a cohort of 31,278 participants. An additional exclusion criterion was incomplete data on CVD (*n* = 4), leaving a total of 31,274 subjects. Data from pregnant individuals were also excluded (*n* = 254), resulting in a final sample size of 31,020 participants for analysis. This rigorous selection process ensured the reliability and validity of our study results (Figure 1).

### 2.2 Definitions of the exposure and outcome variables

In this research, the main variable of exposure examined was eGFR, which is not directly available in the NHANES database. Therefore, we used the Chronic Kidney Disease Epidemiology Collaboration (CKD-EPI) equation, developed by Levey et al. (16). The CKD-EPI equation is formulated as follows: GFR = 141 × min(Scr/*κ*, 1)*α* × max(Scr/*κ*, 1)^ − 1.209 × 0.993^Age × 1.018 [if female] × 1.159 [if black people], where Scr is serum creatinine, *κ* is 0.7 for females and 0.9 for males, *α* is −0.329 for females and −0.411 for males. The min function indicates the minimum of Scr/*κ* or 1, and the max function indicates the maximum of Scr/*κ* or 1.

The outcome variable for this study was cardiovascular disease (CVD). CVD was identified through self-reported diagnoses confirmed by a physician, consistent with criteria used in previous studies (17). Participants were asked whether a doctor or health care professional had diagnosed them with any of the following: Congestive Heart Failure, Coronary Heart Disease, angina pectoris, Myocardial Infarction, or stroke. A “yes” answer to any of these questions classified a participant as having cardiovascular disease.

### 2.3 Covariates

Data were collected on several demographic and health-related factors, including age, gender, race (Mexican American, other Hispanic, non-Hispanic white people, non-Hispanic black people, other), education level (less than high school, high school or equivalent, college or higher), marital status (married/cohabiting, widowed/divorced/separated, never married), and family income-to-poverty ratio. In addition, information was collected on sedentary time (<3 h/day, 3–6 h/day, >6 h/day), smoking status (current, former, never), and alcohol consumption (non-drinker, 0 to <5 drinks/month, 5 to <10 drinks/month, 10+ drinks/month). Hypertension was identified by self-reported diagnosis, use of antihypertensive medication, or if systolic/diastolic blood pressure was ≥130/80 mmHg. Diabetes was defined by self-reported diagnosis, use of insulin or antidiabetic medication, or if fasting plasma glucose was ≥7.0 mmol/L, 2 h postprandial plasma glucose was ≥200 mg/dl, or glycated hemoglobin A1c was ≥6.5%.

### 2.4 Statistical analysis

**Figure 2:**
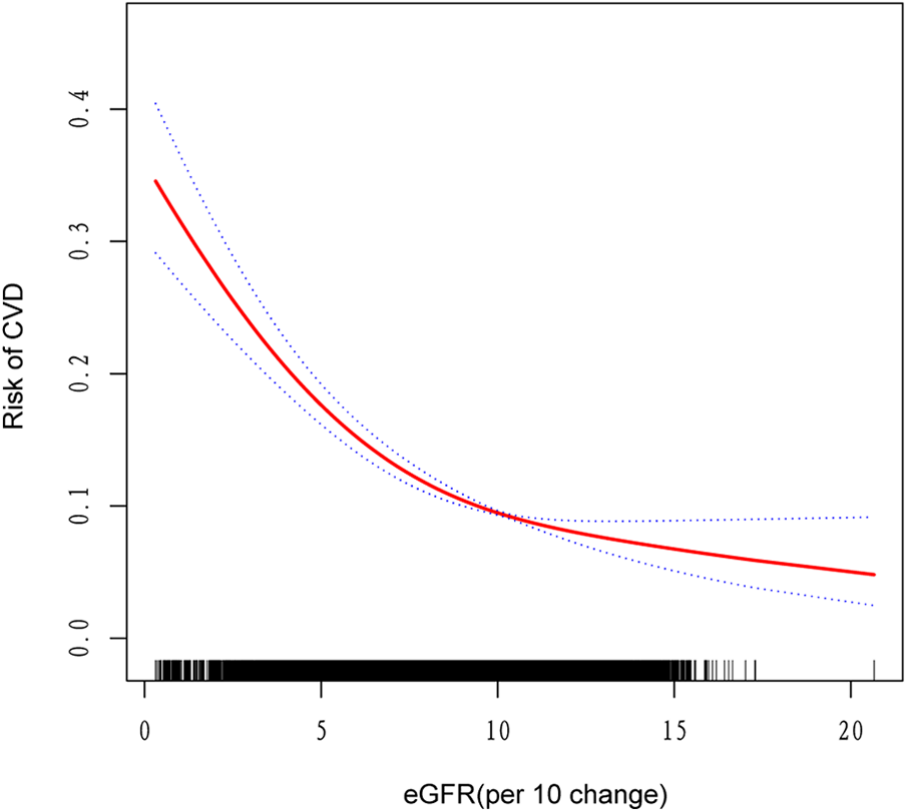
The nonlinear associations between eGFR and risk of CVD. The solid red line represents the smooth curve fit between variables. Blue bands represent the 95% confidence interval from the fit.

Data analysis in this study followed the guidelines of the United States National Center for Health Statistics. Means with 95% confidence intervals (CIs) were reported for continuous variables, and percentages with 95% confidence intervals (CIs) were reported for categorical variables. The purpose of this study was to examine the potential association between eGRF and CVD in selected participants. We used the random forest method to impute missing values of covariates, which can better ensure the integrity and accuracy of the data. We analyzed the continuous variable of eGFR by dividing it into quartiles, as shown in Table 1. Three weighted linear regression models were developed: unadjusted, minimally adjusted, and fully adjusted, as shown in Table 2. To address the issue of multicollinearity, we conducted a multicollinearity test on the indicators in our model using the Variance Inflation Factor (VIF), with the results shown in Supplementary Table S1. The aim was to examine the linear relationship between eGFR and CVD. In addition, the nonlinear relationship between eGFR and CVD was evaluated using a generalized additive model (GAM), as shown in Figure 2. After observing a nonlinear relationship, a two-piecewise linear regression model was performed to calculate the threshold effect of eGFR on CVD in terms of the smoothing curve. When the relationship between eGFR and CVD appears obvious in the smoothed curve, the recursive method automatically calculates the inflection point where maximum model likelihood is used. Table 4 presents a subgroup analysis that was conducted to investigate the association between eGFR and CVD in different subgroups, including sex, age, UACR levels, eGFR levels, diabetes status, anemia status, education level, and PIR. Interaction tests were performed to examine whether these associations were consistent across the various subgroups. All statistical analyses were performed with R, version 4.3.1 (http://www.Rproject.org, The R Foundation) and Empower (R) (www.empowerstats.com; X&Y Solutions, Inc., Boston, MA). Results were considered statistically significant at a two-tailed *P* < 0.05.

**Table 1:** Basic characteristics of study participants stratified by eGFR, weighted.

Variable	eGFR				*P*-value
	Q1	Q2	Q3	Q4	
Age	63.77 (63.23, 64.30)	52.66 (52.12, 53.19)	43.65 (43.20, 44.09)	31.41 (31.08, 31.74)	<0.001
PIR	3.12 (3.04, 3.20)	3.28 (3.19, 3.37)	3.09 (3.00, 3.17)	2.44 (2.36, 2.52)	<0.001
BMI (kg/m^2^)	29.44 (29.23, 29.66)	29.05 (28.83, 29.27)	29.05 (28.81, 29.28)	28.79 (28.50, 29.08)	<0.001
Waist Circumference (cm)	102.27 (101.72, 102.82)	100.11 (99.53, 100.68)	99.02 (98.43, 99.61)	96.07 (95.36, 96.78)	<0.001
eGFR (ml/min/1.73 m^2^)	63.45 (63.09, 63.80)	87.79 (87.63, 87.94)	103.01 (102.88, 103.14)	121.26 (120.97, 121.56)	<0.001
ALT (U/L)	22.93 (22.49, 23.36)	25.47 (24.95, 25.98)	26.42 (25.84, 27.01)	25.72 (25.13, 26.31)	<0.001
AST (U/L)	24.99 (24.61, 25.37)	25.47 (25.04, 25.90)	25.62 (25.11, 26.14)	24.71 (24.24, 25.18)	0.029
Hb (g/dl)	14.01 (13.94, 14.09)	14.39 (14.34, 14.45)	14.35 (14.29, 14.41)	14.06 (14.00, 14.11)	<0.001
UACR (mg/g)	83.42 (70.84, 95.99)	20.91 (17.36, 24.46)	16.24 (14.62, 17.85)	21.62 (18.36, 24.88)	<0.001
Gender, %					<0.001
Man	47.06 (45.77, 48.35)	52.23 (50.81, 53.66)	50.89 (49.46, 52.30)	44.28 (42.76, 45.81)	
Female	52.94 (51.65, 54.23)	47.77 (46.34, 49.19)	49.11 (47.70, 50.54)	55.72 (54.19, 57.24)	
Race, %					<0.001
Mexican American	3.29 (2.53, 4.28)	5.08 (4.06, 6.33)	9.26 (7.76, 11.02)	17.08 (14.49, 20.01)	
Other Hispanic	3.42 (2.76, 4.23)	4.55 (3.66, 5.63)	6.85 (5.73, 8.18)	8.76 (7.55, 10.15)	
Non-Hispanic White People	79.09 (76.62, 81.37)	75.30 (72.59, 77.83)	65.04 (62.03, 67.93)	46.94 (43.34, 50.57)	
Non-Hispanic Black People	8.65 (7.37, 10.14)	7.86 (6.77, 9.11)	9.32 (8.11, 10.69)	17.28 (15.32, 19.43)	
Other Race	5.54 (4.77, 6.43)	7.21 (6.27, 8.27)	9.53 (8.41, 10.79)	9.94 (8.58, 11.48)	
Education Level, %					<0.001
Lower than High School	15.88 (14.30, 17.60)	13.42 (12.11, 14.84)	15.33 (14.01, 16.74)	19.36 (17.79, 21.02)	
High School or Equivalent	23.73 (22.31, 25.21)	22.34 (20.76, 24.01)	22.14 (20.45, 23.93)	23.78 (22.09, 25.55)	
College or Above	60.26 (58.13, 62.34)	64.17 (61.88, 66.40)	62.50 (59.95, 64.98)	56.80 (54.33, 59.23)	
Marital Status, %					<0.001
Married/Cohabiting	62.39 (60.25, 64.48)	68.54 (67.13, 69.92)	65.59 (64.07, 67.09)	55.48 (53.53, 57.40)	
Widowed/Divorced/Separated	30.58 (28.87, 32.35)	20.34 (19.13, 21.62)	15.90 (14.73, 17.16)	7.96 (7.27, 8.71)	
Never Married	7.00 (6.08, 8.05)	11.11 (10.08, 12.23)	18.44 (16.90, 20.09)	36.53 (34.57, 38.54)	
Smoking Status, %					<0.001
Never	53.95 (52.17, 55.72)	54.81 (53.06, 56.55)	52.70 (50.84, 54.56)	61.11 (59.24, 62.94)	
Current	33.64 (32.26, 35.05)	27.52 (26.21, 28.87)	23.65 (21.97, 25.41)	14.24 (12.98, 15.59)	
Former	12.37 (11.30, 13.53)	17.66 (16.33, 19.07)	23.59 (21.87, 25.41)	24.61 (23.22, 26.06)	
Alcohol intake, %					<0.001
Non-drinker	16.97 (15.81, 18.20)	12.11 (11.01, 13.30)	10.29 (9.32, 11.34)	6.75 (5.94, 7.65)	
1 to <5 drinks/month	31.37 (29.84, 32.94)	31.21 (29.50, 32.98)	30.46 (28.79, 32.18)	32.08 (30.64, 33.55)	
5 to <10 drinks/month	6.54 (5.75, 7.44)	8.12 (7.29, 9.04)	9.74 (8.74, 10.84)	10.09 (9.09, 11.19)	
10+ drinks/month	22.21 (20.82, 23.67)	30.46 (28.60, 32.38)	31.64 (29.73, 33.62)	28.96 (27.52, 30.45)	
Sedentary time (hours)					<0.001
<3 h/day	8.61 (7.82, 9.47)	11.69 (10.72, 12.74)	14.40 (13.39, 15.46)	17.96 (16.65, 19.35)	
3–6 h/day	45.82 (43.81, 47.83)	47.51 (45.81, 49.21)	44.79 (43.16, 46.42)	45.20 (43.73, 46.67)	
>6 h/day	44.77 (42.66, 46.90)	40.36 (38.65, 42.10)	40.49 (38.84, 42.17)	36.54 (34.76, 38.35)	
Hypertension, %					<0.001
No	30.75 (29.04, 32.51)	47.42 (45.69, 49.16)	56.59 (54.87, 58.29)	73.44 (72.14, 74.69)	
Yes	69.25 (67.49, 70.96)	52.58 (50.84, 54.31)	43.41 (41.71, 45.13)	26.56 (25.31, 27.86)	
Diabetes, %					<0.001
No	72.31 (70.80, 73.78)	81.49 (80.02, 82.87)	84.18 (83.01, 85.29)	87.17 (86.15, 88.13)	
Yes	27.69 (26.22, 29.20)	18.51 (17.13, 19.98)	15.82 (14.71, 16.99)	12.83 (11.87, 13.85)	
CVD, %					<0.001
No	79.13 (77.75, 80.44)	91.23 (90.45, 91.96)	95.64 (94.96, 96.22)	98.02 (97.61, 98.35)	
Yes	20.87 (19.56, 22.25)	8.77 (8.04, 9.55)	4.36 (3.78, 5.04)	1.98 (1.65, 2.39)	
Amemia, %					<0.001
No	88.45 (87.27, 89.53)	94.96 (94.19, 95.63)	94.61 (93.77, 95.34)	92.01 (91.13, 92.81)	
Yes	11.35 (10.30, 12.49)	4.75 (4.11, 5.48)	5.11 (4.43, 5.88)	7.87 (7.07, 8.75)	

eGFR, Estimated Glomerular Filtration Rate; BMI, Body Mass Index; PIR, Ratio of Family Income to Poverty; CVD, Cardiovascular Disease; Hb, Hemoglobin; UACR, Urine Albumin Creatinine Ratio; ALT, Alanine Aminotransferase; AST, Aspartate Aminotransferase.

Numbers that do not add up to 100% are attributable to missing data.

Mean (95% CI) for continuous variables: the *P* value was calculated by the weighted linear regression model.

Percentages (95% CI) for categorical variables: the *P* value was calculated by the weighted chi-square test.

**Table 2:** Associations of eGFR with CVD by the weighted linear model.

eGFR (per 10 change)	Model 1	Model 2	Model 3
	OR (95% CI)	OR (95% CI)	OR (95% CI)
Continuous	0.66 (0.64, 0.67)	0.85 (0.83, 0.88)	0.89 (0.86, 0.91)
eGFR quartiles			
Q1 (<7.88)	Reference	Reference	Reference
Q2 (7.88–9.57)	0.36 (0.33, 0.41)	0.67 (0.59, 0.76)	0.74 (0.64, 0.84)
Q3 (9.57–11.09)	0.17 (0.15, 0.20)	0.65 (0.53, 0.80)	0.61 (0.50, 0.74)
Q4 (>11.09)	0.08 (0.06, 0.09)	0.73 (0.57, 0.96)	0.69 (0.54, 0.90)
*P* for trend	<0.001	<0.001	<0.001

Model 1: adjust for: none.

Model 2: adjust for: gender, age, and race.

Model 3: adjust for: gender, age, race, education level, marital status, ratio of family income to poverty, smoking status, alcohol intake, Sedentary time, hypertension, diabetes, AST, ALT,BMI, Waist Circumference, Hb.

## 3 Results

### 3.1 Basic characteristics

Table 1 shows the baseline characteristics of the study participants stratified by eGFR quartiles. The data show significant differences (*P* < 0.001) across eGFR quartiles for all variables examined except AST (*P* = 0.029). Age and waist circumference showed a decreasing trend with increasing eGFR quartiles. BMI and PIR also varied significantly across quartiles, with the lowest values observed in Q4. The proportion of women was higher in Q4 compared to other quartiles. The distribution of race varied significantly, with the highest proportions of Mexican Americans and non-Hispanic black people in Q4 and the highest proportions of non-Hispanic white people in Q1. Education level, marital status, smoking status, and alcohol consumption also varied significantly across eGFR quartiles. The proportion of participants with less than a high school education and those who had never been married was highest in Q4. The proportion of current smokers decreased from Q1 to Q4, whereas the proportion of former smokers increased. Non-drinkers were more common in Q1, while those consuming 10+ drinks/month were more common in Q3. Sedentary time showed a decreasing trend with increasing eGFR quartiles. The prevalence of hypertension, diabetes and cardiovascular disease (CVD) decreased significantly from Q1 to Q4. Additionally, ALT levels were highest in Q3, hemoglobin levels were highest in Q2 and Q3, UACR was notably higher in Q1 compared to other quartiles, and the prevalence of anemia was highest in Q1 and lowest in Q2.

### 3.2 Association between eGFR and CVD

Table 2 shows the associations between eGFR and CVD using weighted linear regression models. The results show a significant inverse relationship between eGFR and CVD risk, with lower eGFR values associated with higher odds of CVD. In the unadjusted model (model 1), a 10-unit increase in eGFR was associated with a 34% lower odds of CVD (OR: 0.66, 95% CI: 0.64–0.67, *P* < 0.001). This association remained significant but was attenuated after adjustment for demographic factors (gender, age, and race) in model 2 (OR: 0.85, 95% CI: 0.83–0.88, *P* < 0.001) and after further adjustment for socioeconomic status, lifestyle factors, and comorbidities in model 3 (OR: 0.89, 95% CI: 0.86–0.91, *P* < 0.001). A dose-response relationship was observed when analyzing eGFR as quartiles. Compared to the lowest eGFR quartile (Q1, < 7.88), higher eGFR quartiles were associated with progressively lower odds of CVD. In the fully adjusted model (model 3), the odds of CVD were 26% lower in Q2 (OR: 0.74, 95% CI: 0.64–0.84), 39% lower in Q3 (OR: 0.61, 95% CI: 0.50–0.74), and 31% lower in Q4 (OR: 0.69, 95% CI: 0.54–0.90) compared to Q1 (all *P* < 0.001 for trend).

### 3.3 Curve fitting and threshold effect analysis

Table 3 presents the results of a threshold effect analysis examining the relationship between estimated eGFR and CVD risk. The analysis identified an inflection point at an eGFR value of 99.3 ml/min/1.73 m², indicating a non-linear relationship between eGFR and cardiovascular disease (CVD) risk. Below the inflection point (eGFR < 99.3 ml/min/1.73 m²), a significant inverse relationship was observed. For each 10-unit increase in eGFR, the adjusted odds of CVD decreased by 13% (OR: 0.87, 95% CI: 0.84–0.90, *P* < 0.001). This finding suggests that among individuals with eGFR less than 99.3 ml/min/1.73 m², declining renal function is strongly associated with increased risk of CVD. In contrast, above the inflection point (eGFR > 99.3 ml/min/1.73 m²), no significant association was found between eGFR and CVD risk (OR: 1.04, 95% CI: 0.90–1.20, *P* = 0.60), indicating a threshold effect, where further increases in eGFR beyond this point do not confer additional protection against CVD. The log-likelihood ratio test *P*-value was 0.002, supporting the non-linear relationship between eGFR and CVD risk.

**Table 3:** Threshold effect analysis of eGFR and CVD.

eGFR (per 10 change)	Adjusted OR (95% CI)	*P*-value
Inflection point	9.93	
eGFR < Inflection point	0.87 (0.84, 0.90)	<0.001
eGFR > Inflection point	1.04 (0.90, 1.20)	0.60
Log-likelihood ratio		0.002

Gender, age, race, education level, marital status, the ratio of family income to poverty, smoking status, alcohol intake, Sedentary time, hypertension, diabetes, AST, ALT, BMI, Waist circumference and Hb were adjusted.

Furthermore, we utilized a generalized additive model to validate the non-linear association between eGFR and CVD, confirming a negative correlation as depicted in Figure 2.

### 3.4 Subgroup analyses

Subgroup analyses and interaction tests were conducted to assess whether the association between eGFR and cardiovascular disease (CVD) risk was consistent across different population subgroups (Table 4). Our results revealed significant interactions for eGFR categories (*P* for interaction = 0.02) and anemia status (*P* for interaction = 0.009). For eGFR categories, the association between eGFR and CVD risk varied considerably. In participants with eGFR ≥90 ml/min/1.73 m^2^, the odds ratio (OR) for CVD was 0.74 (95% CI: 0.67–0.81) per 10 ml/min/1.73 m^2^ increase in eGFR. However, for those with eGFR <15 ml/min/1.73 m^2^, the OR increased dramatically to 4.26 (95% CI: 0.61–29.95), albeit with a wide confidence interval. Regarding anemia status, participants without anemia showed a stronger inverse association between eGFR and CVD risk (OR: 0.74, 95% CI: 0.71–0.76) compared to those with anemia (OR: 0.80, 95% CI: 0.76–0.85). Additionally, we observed a significant interaction for PIR categories (*P* for interaction <0.001). The association between eGFR and CVD risk was strongest in the highest PIR group (>350%, OR: 0.70, 95% CI: 0.67–0.75) and weakest in the lowest PIR group (<130%, OR: 0.81, 95% CI: 0.78–0.84). No significant interactions were found for sex, age, UACR, diabetes status, or education level (all *P* for interaction >0.05).

**Table 4:** Subgroup analysis of the association between eGFR (per 10 change) and CVD.

Subgroup	Risk of CVD [OR (95% CI)]	*P* for interaction
Gender		0.318
Male	0.87 (0.84, 0.91)	
Female	0.89 (0.86, 0.93)	
Age		0.501
<60 years	0.81 (0.78, 0.84)	
≥60 years	0.82 (0.79, 0.86)	
UACR		0.328
<30 mg/g	0.89 (0.86, 0.93)	
30–300 mg/g	0.85 (0.81, 0.90)	
>300 mg/g	0.90 (0.82, 1.00)	
eGFR		0.024
≥90 ml/min/1.73 m^2^	0.74 (0.67, 0.81)	
60–89 ml/min/1.73 m^2^	0.83 (0.76, 0.91)	
30–59 ml/min/1.73 m^2^	0.77 (0.66, 0.90)	
15–29 ml/min/1.73 m^2^	2.05 (0.80, 5.25)	
<15 ml/min/1.73 m^2^	4.26 (0.61, 29.95)	
Diabetes, (%)		0.214
Yes	0.90 (0.86, 0.94)	
No	0.87 (0.84, 0.90)	
Anemia, (%)		0.009
Yes	0.80 (0.76, 0.85)	
No	0.74 (0.71, 0.76)	
Education level		0.136
Lower than High School	0.78 (0.75, 0.81)	
High School or Equivalent	0.76 (0.73, 0.79)	
College or Above	0.74 (0.71, 0.77)	
PIR		<0.001
<130%	0.81 (0.78, 0.84)	
130%–350%	0.74 (0.71, 0.77)	
>350%	0.70 (0.67, 0.75)	

eGFR, Estimated Glomerular Filtration Rate; PIR, Ratio of Family Income to Poverty; CVD, Cardiovascular Disease; ALT, Alanine Aminotransferase; AST, Aspartate Aminotransferase; UACR, Urine Albumin Creatinine Ratio.

Gender, age, race, education level, marital status, PIR, smoking status, alcohol intake, Sedentary time, hypertension, diabetes, AST, ALT, BMI, Waist circumference and Hb were adjusted except for the subgroup variable.

## 4 Discussion

This study used data from the National Health and Nutrition Examination Survey (NHANES) in the United States to examine the association between eGFR and CVD risk. By analyzing information from 31,020 patients, we found that eGFR and CVD risk had a non-linear negative association. This study discovered the presence of a threshold and saturation effect between eGFR and CVD risk. Moreover, the impact of eGFR changes on CVD risk varied significantly across different eGFR groups. Notably, anemia status and PIR) levels showed significant modifying effects on the association between eGFR and CVD risk. Specifically, among individuals with anemia and those in higher income groups, each 10 ml/min/1.73 m^2^ increase in eGFR was associated with a greater reduction in CVD risk. These findings suggest that factors such as anemia and socioeconomic status should be considered when assessing the relationship between kidney function and cardiovascular disease risk.

It is important to note that our findings varied across different stages of chronic kidney disease (CKD). For less severe CKD stages (eGFR ≥30 ml/min/1.73 m^2^), we consistently observed a negative association between eGFR and CVD risk, suggesting that higher eGFR levels are associated with lower CVD risk in these groups. However, for more advanced CKD stages (eGFR <30 ml/min/1.73 m^2^), the estimates showed wide confidence intervals, indicating a lack of precision likely due to smaller sample sizes in these groups.The apparent positive association observed in the more advanced CKD stages should be interpreted with caution due to these wide confidence intervals. These results highlight the need for larger studies focusing specifically on advanced CKD patients to more accurately assess the relationship between eGFR and CVD risk in these stages. In future research, it will be crucial to gather more data on patients with advanced CKD to better understand the eGFR-CVD risk relationship in this population.

A study of 13,029 participants from the ARIC cohort found that a steeper decline in eGFR was associated with a higher risk of coronary heart disease and all-cause mortality, even after adjusting for baseline eGFR and other risk factors. An increase in eGFR among those with chronic kidney disease was also linked to increased mortality risk (18). Mathisen et al. measured GFR by iohexol clearance in a general population sample aged 50–62 years and found that eGFR, calculated using creatinine or cystatin C, was associated with traditional cardiovascular risk factors, even after adjusting for measured GFR. This suggests that eGFR calculations can be influenced by factors other than true GFR (19). Guo et al. conducted a prospective cohort study of 37,691 participants from the Kailuan Study and found a significant association between annual eGFR decline and increased risk of all-cause mortality and CVD, independent of baseline eGFR and other known risk factors (20). Ramezankhani et al. conducted a prospective cohort study in the Tehran Lipid and Glucose Study involving 2,873 participants and demonstrated that eGFR slope was associated with an increased risk of cardiovascular events in their sample (21). In another study led by Dupuis et al, a large prospective cohort of 9,515 healthy individuals in Quebec demonstrated a strong association between extreme eGFR decline and increased risk of cardiovascular events, underscoring the importance of monitoring eGFR changes in individuals with normal to mildly reduced kidney function (22). Finally, Suchy-Dicey et al. in the Strong Heart Study followed approximately 20,000 Native Americans over a decade and showed that even in those with normal to slightly reduced eGFR, a decline in eGFR was significantly associated with an increased 10-year risk of atherosclerotic cardiovascular disease (23). Grams et al. conducted an individual-participant data meta-analysis to evaluate the associations between eGFR, calculated using the CKD-EPI 2021 equations, and cardiovascular disease and mortality, and showed that lower eGFR based on creatinine and cystatin C was negatively associated with cardiovascular disease and cardiovascular mortality (1). Our study's findings align with prior research demonstrating a negative link between eGFR and CVD risk, and further verify the influence of confounding factors on this association. However, our study identified threshold and saturation effects between eGFR and CVD risk. This novel finding provides important insights into understanding the nonlinear association between the two. Differences between studies may be due to differences in population characteristics and study design.

The association between eGFR and CVD is complex and can be elucidated by various mechanisms. As eGFR declines, renal function deteriorates, leading to the accumulation of toxins, metabolic wastes, endothelial damage, endothelial dysfunction, systemic inflammation, and increased oxidative stress (24–26). These factors collectively contribute to the development of atherosclerosis, a primary pathological basis of CVD (24). Additionally, decreased renal function can lead to elevated blood pressure, abnormal lipid metabolism, calcium and phosphorus imbalances, autonomic nervous system dysfunction, and anemia (27). Increased blood pressure can have detrimental effects on kidney health, potentially leading to glomerular hypertension, accelerated glomerular injury, and decreased renal function, which can contribute to the development and progression of CKD (28). Dyslipidemia consistently manifests in the development of CKD, even during the initial stages of reduced eGFR. The primary characteristics of dyslipidemia in CKD are elevated triglyceride levels and reduced high-density lipoprotein cholesterol levels (29). Inflammation plays a crucial role in the relationship between CKD and CVD, influencing the development of cardiovascular complications and serving as a prognostic indicator (30, 31). Additionally, the presence of renal dysfunction may serve as a marker for cardiovascular dysautonomia, a characteristic feature of prodromal Parkinson's disease, suggesting a potential link between renal dysfunction and cardiovascular autonomic disturbances (32). The interactions and overlaps among these factors further strengthen the connection between renal insufficiency and CVD.

This study highlights the association between declining kidney function and increased risk of CVD, suggesting that clinicians need to closely monitor and manage CVD risk factors in patients with reduced kidney function, especially in high-risk individuals with an eGFR (per 10 change) below 9.93. Furthermore, the results indicate that raising eGFR above this threshold may not provide additional cardiovascular benefits, which has important implications for the development of individualized renal and cardiovascular management strategies.

A major strength of this study is the use of a large, nationally representative sample from NHANES, which allows the generalizability of the results to the adult population in the United States. The complex, multistage probability sampling design of NHANES and the application of appropriate sample weights ensure the representativeness of the results. In addition, we controlled for a wide range of potential confounding factors, thereby increasing the robustness of the results. This study also examined the nonlinear relationship between eGFR and CVD, providing new insights into the complex relationship between kidney function and cardiovascular health.

Nevertheless, it is important to recognize several limitations of this study. First, the cross-sectional nature of NHANES does not allow for the determination of a causal link between eGFR and CVD. Second, despite controlling for multiple confounders, the possibility of residual confounding cannot be completely excluded. Third, the estimation of GFR using creatinine-based equations has inherent limitations that may affect the accuracy of eGFR estimation.Fourth,our study was limited by the inaccessibility of NHANES urban-rural data, preventing analysis of these population differences.We emphasize the critical importance of examining urban-rural disparities in future CKD research. Fifth, our cross-sectional design limited the ability to observe dynamic changes in eGFR-CVD risk relationships.

Future prospective cohort studies are needed to validate the causal relationship between eGFR and CVD risk, explore the potential mechanisms underlying the association, and develop and validate CVD risk assessment tools and management strategies tailored to different eGFR levels. Future studies should either design targeted urban-rural surveys or collaborate with institutions having access to restricted NHANES data. Future longitudinal studies are crucial to explore these changes during nephropathy reversal, potentially elucidating the impact of improved kidney function on cardiovascular risk.

## 5 Conclusion

In conclusion, this study demonstrates a non-linear inverse association between eGFR and CVD risk, with evidence of a threshold and saturation effect. Management of CVD risk in patients with impaired renal function requires an individualized approach. These findings have important implications for clinical practice and public health policy, and contribute to the optimization of cardiovascular health management in patients with chronic kidney disease.

## Data Availability

The raw data supporting the conclusions of this article will be made available by the authors, without undue reservation.
