# Supplementary material for: The nonlinear relationship between estimated glomerular filtration rate and cardiovascular disease in US adults: a cross-sectional study from NHANES 2007–2018
Source: Front Cardiovasc Med. 2024 Nov 22;11:1417926. doi: 10.3389/fcvm.2024.1417926 (PMC11621053; doi:10.3389/fcvm.2024.1417926)
Supplement: Supplementary file 1 [file Table1.docx]

**Additional methods:**

Detailed Explanation of Curve Fitting and Threshold Effect Analysis

Curve Fitting

To fit a curve to the data, we employ the Generalized Additive Models (GAM) using the mgcv package in R. Here's the detailed method:

Method: If the dependent variable  Y is continuous or binary, we use the gam() function from the mgcv package.

Spline Functions: The curve fitting component is defined using the s() function within gam().

Degrees of Freedom: We set the degrees of freedom to 3. The mgcv package typically creates thin plate regression splines, with a default maximum of 10 degrees of freedom. The penalty for spline smoothness is optimized using generalized cross validation by default.

Reference: Perperoglou, A., Sauerbrei, W., Abrahamowicz, M., et al. (2019). A review of spline function procedures in R. BMC Medical Research Methodology, 19(46).  <https://doi.org/10.1186/s12874-019-0666-3>

Threshold Effect or Saturation Effect Analysis

To analyze potential threshold or saturation effects, we use the following approach:

Smooth Curve Fitting: Initially, we fit a smooth curve to examine if the independent variable partitions into distinct intervals.

Segmented Regression: We then apply segmented regression (piece-wise regression) by fitting separate line segments to each interval.

Log-likelihood Ratio Test: To establish the presence of a threshold, we compare a single-line (non-segmented) model to a segmented regression model using a log-likelihood ratio test. The existence of a threshold is supported if the segmented model has a higher likelihood.

Inflection Point Determination: The model that provides the maximum likelihood determines the inflection point connecting the segments. This is identified through a two-step recursive method:

Step 1: We narrow down the inflection point using a 10 percentile range of the independent variable. From 5% to 95%, incrementing by 5%, we test 19 segmented regression models with each percentile point as the potential inflection point. The model with the highest likelihood among these determines the initial inflection range, defining Kmin​ and Kmax​.

Step 2: We further refine the inflection point within the Kmin​ andKmax​ range using a recursive method. This involves:

Running three models with inflection points at the 25th percentile (Q1), 50th percentile (Q2), and 75th percentile (Q3) within the Kmin​ to Kmax​ range.

Identifying which quartile point yields the model with the highest likelihood and updating  Kmin​ and  Kmax​ to a narrower range of ±25% of this quartile point.

Repeating this process to halve the range until the exact value of the independent variable that serves as the inflection point is identified, maximizing the segmented model's likelihood.

Table S1. Collinearity Check: Variance Inflation Factor (VIF) Selection

| **Variable** | VIF |
| --- | --- |
| Waist circumference | 7.8 |
| BMI | 7.4 |
| Age | 2.7 |
| ALT | 2.7 |
| AST | 2.5 |
| EGFR10 | 2.1 |
| Gender | 1.7 |
| Hemoglobin | 1.5 |
| Education level | 1.4 |
| Hypertension | 1.4 |
| PIR | 1.4 |
| Alcohol status | 1.3 |
| Race | 1.1 |
| Marital status | 1.1 |
| Smoking status | 1.1 |
| Sedentary time | 1.1 |
| Diabetes | 1.1 |

Note: VIF = Variance Inflation Factor; BMI = Body Mass Index; ALT = Alanine Aminotransferase; AST = Aspartate Aminotransferase; EGFR10 = Estimated Glomerular Filtration Rate (per 10 change); PIR = Ratio of Family Income to Poverty
